# Systems biology of unfolded protein response in recombinant CHO cells

**DOI:** 10.1186/1753-6561-7-S6-P67

**Published:** 2013-12-04

**Authors:** Kamal Prashad Segar, Vikas Chandrawanshi, Sarika Mehra

**Affiliations:** 1 Department of Chemical Engineering, Indian Institute of Technology Bombay, Mumbai - 400076, India

## Background

Productivity of recombinant therapeutics is a coordinated effort of multiple pathways in the cell [1]. The protein processing pathway in endoplasmic reticulum has been the target of many cell engineering studies but with mixed results [2]. We have observed the induction of UPR genes in recombinant CHO cells (data not shown). In this work, we attempt to increase their productivity further by inducing ER stress using a known UPR inducer.

## Materials and methods

### Cell culture

Suspension CHO cells secreting anti rhesus IgG were grown in a media containing 50% PF-CHO (Hyclone) and 50% CDCHO (Invitrogen) supplemented with 4 mM L-Glutamine (Invitrogen), 0.10% Pluronic (Invitrogen), 600 μg/ml G418 (Sigma) and 250 nM Methotrexate (Sigma) in a total culture volume of 20 ml. All cultures were run in replicates in 125 ml Erlenmeyer flasks (Corning). Cells were treated with tunicamycin (Sigma) for 12 hours and were harvested for RNA isolation. Cell densities and viabilities were determined by a hemocytometer using the tryphan blue exclusion method.

### Quantitative real time PCR

Primers were designed based on consensus sequences from human, mouse and rat and checked against the CHO genome database wherever available. Total RNA was isolated using Tri reagent (Sigma) and converted to cDNA using the Reverse Transcription kit (Thermo). 100 ng of cDNA was used for qPCR to quantify the mRNA levels of different UPR genes with Actin as the house keeping genes following the ΔΔCT method.

### Antibody quantification

Antibody titres were quantified using the protocol as described earlier by Chusainow et.al.,[3] and their specific productivities (qP) were also calculated.

## Results

Induction of different ER stress genes was observed at peak productivities in these recombinant CHO cell lines (data not shown). Therefore, we hypothesized that increasing the ER stress to higher levels may have an additive effect on IgG productivity in these cell lines. Tunicamycin a known ER stress inducer was used to induce ER stress in these cells. CHO cells were treated with tunicamycin (2.5 mM) for 12 hours and harvested for RNA isolation. qPCR was performed to quantitate the expression levels of different ER stress genes. IgG HC and LC mRNA were also quantified and their fold changes were also calculated. IgG titers in the supernatant were quantified using ELISA.

**Figure 1:**
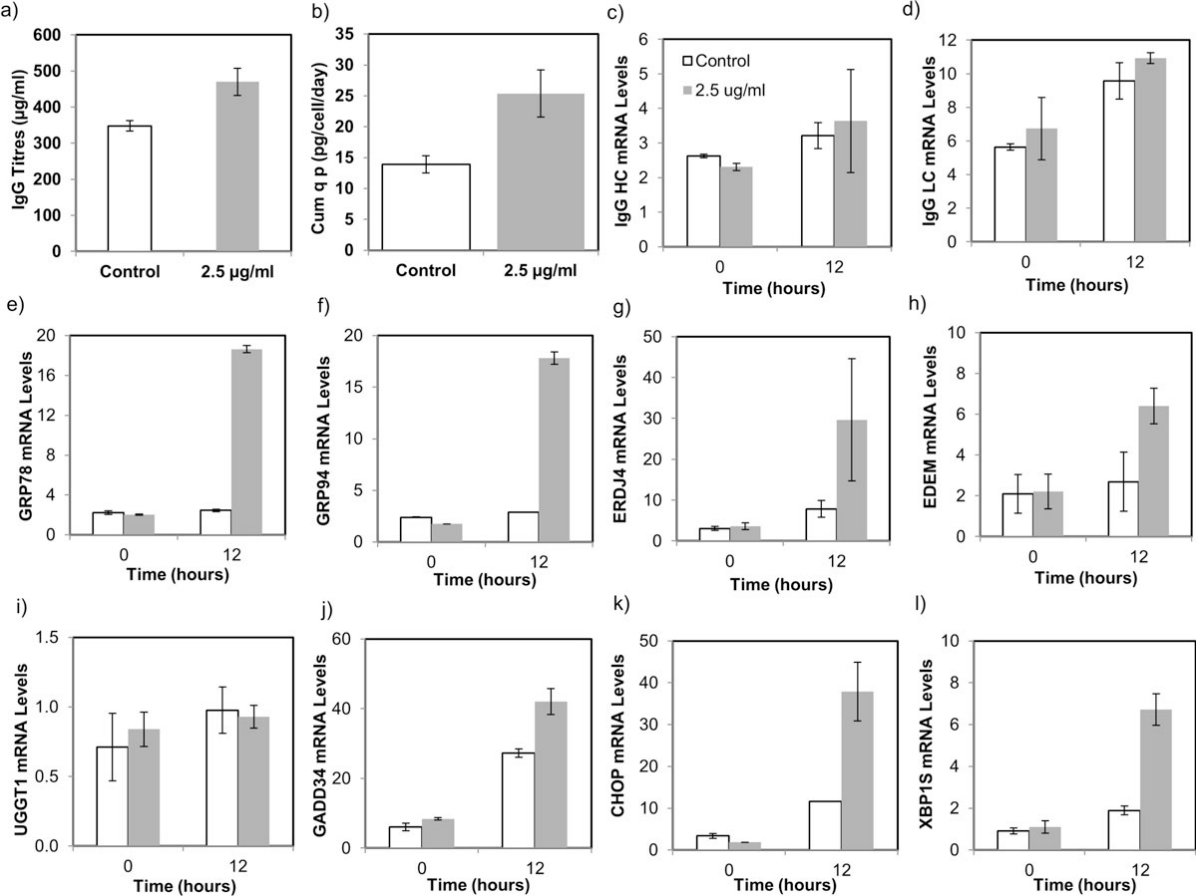
**Effect of Tunicamycin on the IgG titres, productivities and mRNA levels of different UPR genes**.

The IgG titers and cumulative productivities in the tunicamycin treated and control cells are presented in Figures 1a and 1b. 12 hours post-treatment with tunicamycin, the IgG titers increased to 460 μg/ml. Productivity in treated cells was found to be 25 pg/cell/day, corresponding to a 1.7 fold increase compared to control cells. Interestingly, both the IgG HC and LC mRNA were not induced in treated cells (Figure 1c, d). To elucidate the role of UPR pathway in the observed increase in productivity, expression of many chaperones and UPR genes was measured. In response to tunicamycin, chaperones including GRP78 and GRP94 were induced to a maximum of 17-fold (Figures 1e, f). Co-chaperone ERDJ4, involved in the translocation of nascent proteins inside ER and activation of ERAD pathway [4], was also induced in response to tunicamycin treatment indicating increase in ER load. Figure 1g and 1h show the mRNA profiles of ERDJ4 and EDEM in control and treated cells. No significant difference in expression of UGGT1 mRNA was observed, suggesting that there may be negligible mis-folded proteins (Figure 1i) which can be recycled for refolding while most of them are continuously degraded by the ERAD machinery. Highly active transcription factors of the UPR pathway viz., GADD34, CHOP and XBP1s were also induced in response to tunicamycin treatment. Figures 1j-1l show the mRNA profiles of different UPR genes. GADD34 was induced to 38-folds on treatment while CHOP mRNA induced to about 30-folds. Spliced XBP1 mRNA was also induced to a maximum of 5.5-folds in treated cells leading to increased expression of GRP78 mRNA.

## Conclusion

Engineering cells towards high productivity by exploiting their cellular pathways has been gaining importance recently in biopharmaceutical industries. The unfolded protein response (UPR) pathway has also been targeted to develop a high producing clone. However, the results from previous engineering studies on this pathway are either cell line or product dependent.

In this study, with prior knowledge on the induction of different UPR genes at peak productivities, we attempted to increase productivity by increasing ER stress using a known UPR inducer, tunicamycin. Tunicamycin induced the expression of chaperones and key UPR transcription factors including GADD34 and XBP1s mRNA.

Increase in the levels of GRP78 and GRP94 mRNA with no change in the levels of the UGGT1 mRNA suggests that the treated cells may possess a highly active folding pathway. Increase in the productivities with no change in the levels of IgG HC and LC mRNA support our hypothesis of an increased folding capacity in treated cells. Hence, we suggest that the UPR pathway can be modulated to increase the productivity.
